# Supplementary material for: Chimeric Vesicular Stomatitis Virus Bearing Western Equine Encephalitis Virus Envelope Proteins E2-E1 Is a Suitable Surrogate for Western Equine Encephalitis Virus in a Plaque Reduction Neutralization Test
Source: Viruses. 2025 Jul 31;17(8):1067. doi: 10.3390/v17081067 (PMC12390645; doi:10.3390/v17081067)
Supplement: Supplementary file 1 [file viruses-17-01067-s001.zip › viruses-3765623-supplementary.pdf]

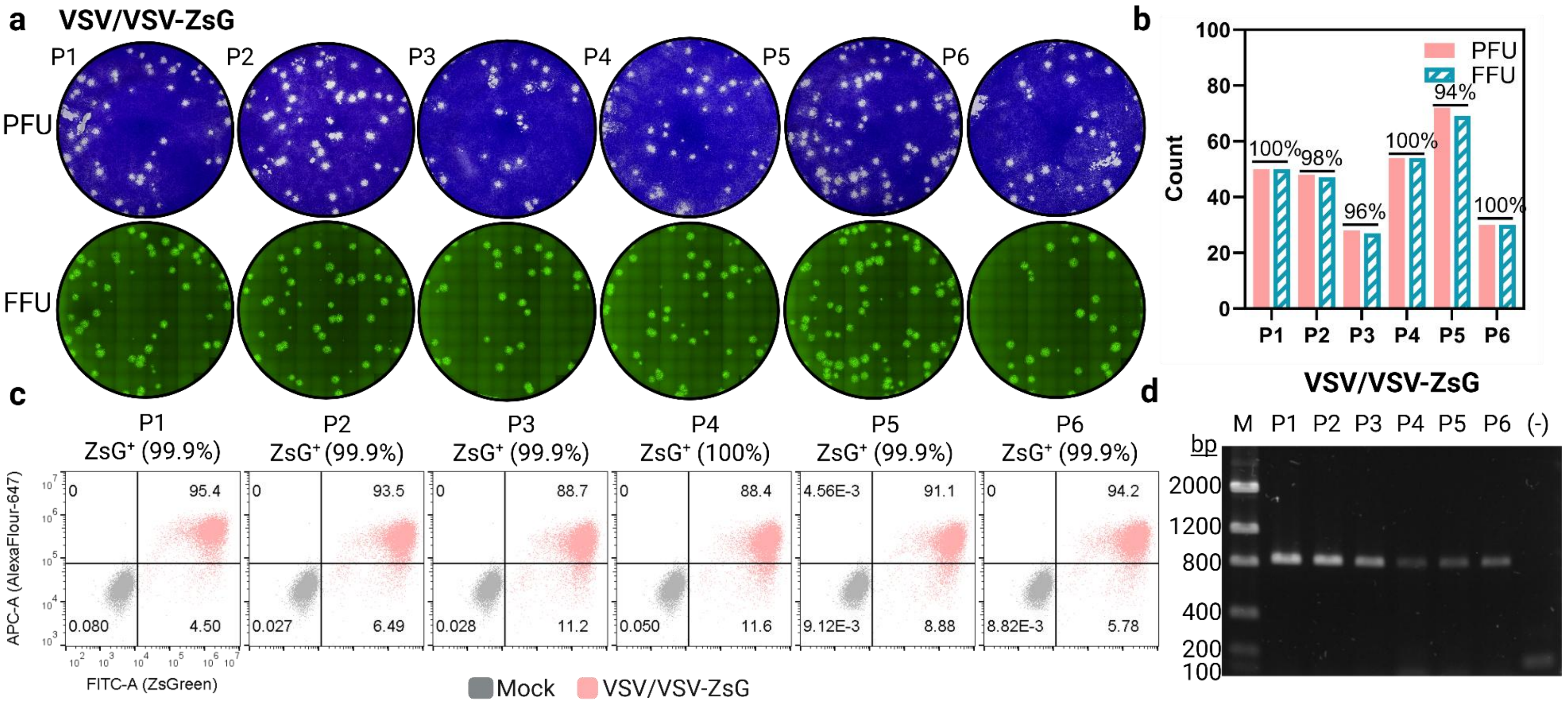

Supplemental Figure S1. ZsGreen stability in chimeric VSV/VSV-ZsG with repeated passaging in Vero cells (**a**) Comparison of PFUs (visual) to FFU (ZsGreen) in Vero cells across six passages (**b**) Enumeration of PFUs to FFU in each well (**c**), FACs analysis of ZsGreen expression (MFI) versus cells collected at 12 hpi stained for VSV-M (AlexaFour-647) in Vero cells infected with a high MOI (1-2) of VSV/VSV-ZsG (pink) using stocks from each passage. Cells were gated for the bulk population, then singlets, with FITC-A and APC-A gates selected from uninfected Vero cells stained with both primary and secondary antibodies (grey population) (**d**), PCR amplicons of genomic position 5 using a one-step RT-PCR reaction with viral RNA from P1-P6 stocks as template and chimeric VSV/VSV without ZsGreen as a negative (-) control.

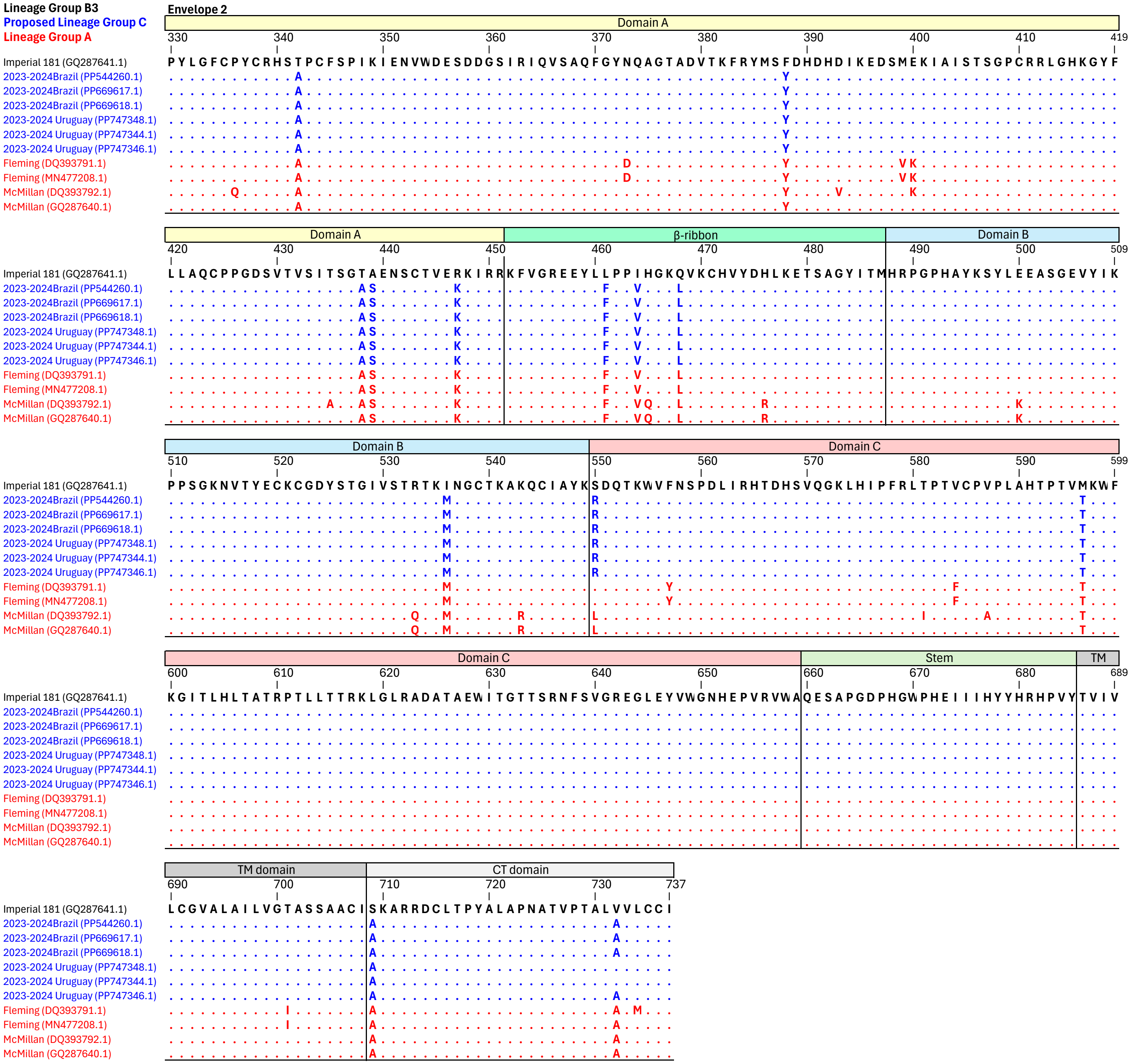

Supplemental Figure S2. Alignment of E2 among WEEV lineages.

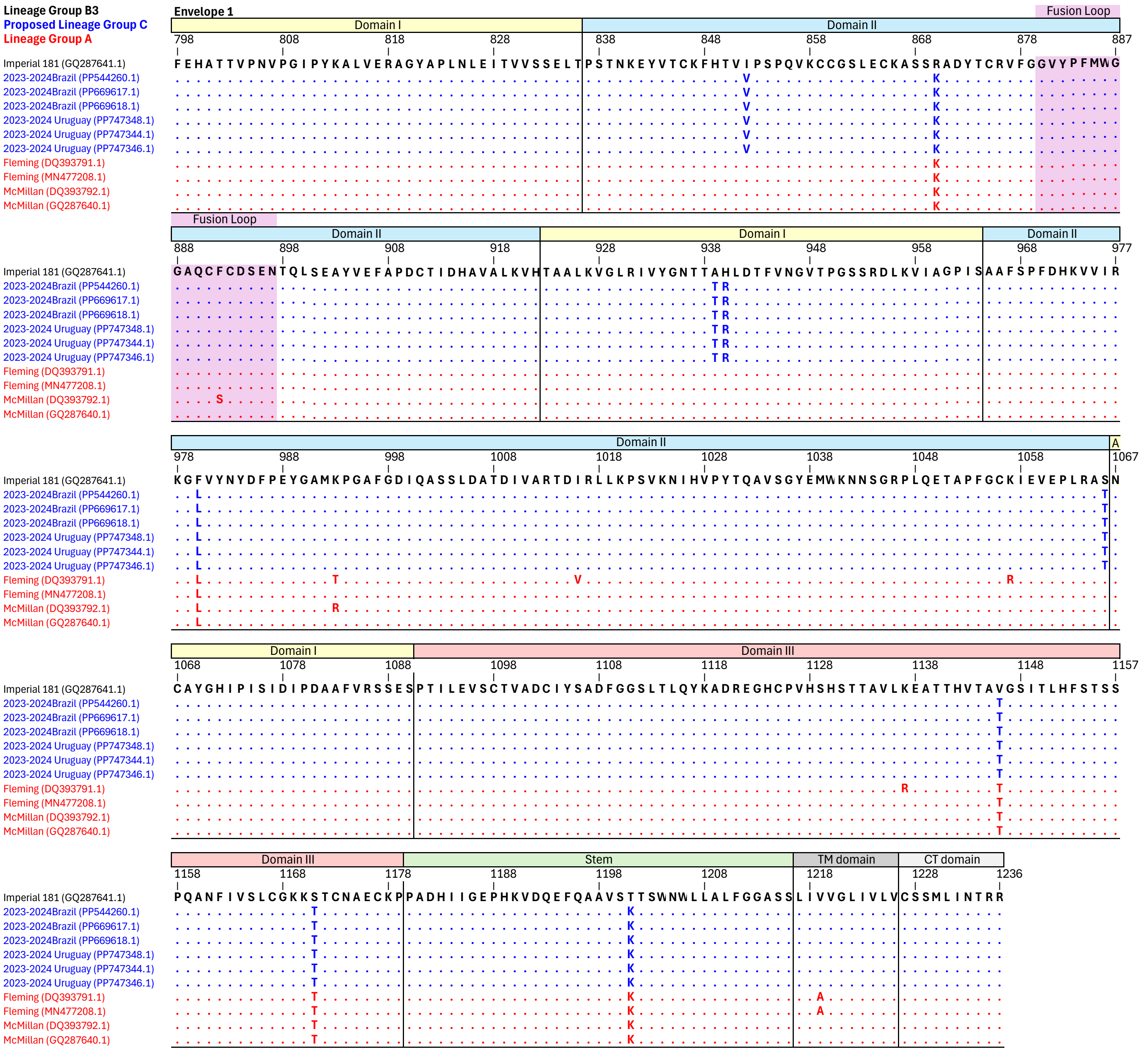

Supplemental Figure S3. Alignment of E1 among WEEV lineages.
